# Hirschsprung’s Disease Presenting as Neonatal Appendicitis

**Published:** 2013-04-01

**Authors:** L Sahnoun, M Kitar, K Maazoun, A Ksia, J Chahed, M Mekki, I Krichen, M Belghith, A Nouri

**Affiliations:** Department of Pediatric Surgery, Fattouma Bourguiba Hospital, Monastir, Tunisia

**Dear Sir**

Acute appendicitis, a common childhood abdominal emergency, is rare in neonates. Non-specific presenting symptoms often result in missed or delayed diagnosis that leads to high morbidity and mortality [1-4]. 

We report a case of a full-term infant girl who presented to us with diarrhea and vomiting for 3 days at the age of 30 days. At presentation, she was dehydrated. The abdomen was unremarkable; hernial sites were normal. WBC at admission was 5000/mm3. Intravenous fluids and antibiotics were started. Three days later, she developed fever (38.10 C), bilious vomiting and bleeding per rectum. Abdomen was still unremarkable. The chest and abdominal radiographs showed no bowel gas and no free air. The diagnosis of high intestinal obstruction due to midgut volvulus was suspected. However, ultrasonography didn’t show any signs of malrotation. The bowel obstruction was thought to result from internal hernia.

Laparotomy revealed acute appendicitis; minimal reactive peritoneal fluid was also present. Appendicectomy was done. Rectal biopsy was performed to rule out underlying Hirschsprung’s disease. The bacteriologic examination of peritoneal fluid was negative. Histopathologic examination revealed necrosis and inflammatory infiltration with neutrophils of appendiceal mucosa; muscularis and serosa were normal. Ganglion cells were identified within the appendix, but were absent in the rectal biopsy. She succumbed to sepsis and multi-organ failure in the immediate post-operative period. 

Neonatal appendicitis has predilection for boys (M:F::3:1), and premature neonates (30%-50% of cases) [1]. There are 2 common presentations. In 1/3 of the cases, it is due to incarceration of appendix in the inguinal hernia. In rest of the cases, the polymorphism of the presenting symptoms could pose a diagnostic challenge [2]. All these non-specific symptoms, associated with the rarity of this disease result in a significant delay in diagnosis and consequently a high mortality rate of 20-25% [3]. 

Two major theories have been proposed to explain intra-abdominal neonatal appendicitis- i) neonatal appendicitis is a localized form of necrotizing enterocolitis, and ii) obstructive caecal distension due to underlying Hirschsprung’s disease, or less frequently with meconium plug syndrome, leads to appendicitis and appendeceal perforation [2]. Our patient probably had Hirschsprung’s related enterocolitis that afflicted appendix too.

## Footnotes

**Source of Support:** Nil

**Conflict of Interest:** None

## References

[1] ( 1995). Dessanti A, Porcu A, Scanu AM, Dettori G. Neonatal acute appendicitis in an inguinal hernia.. Pediatr Surg Int..

[2] ( 2011). Schwartz KL, Gilad E, Sigalet D, Yu W, Wong AL. Neonatal acute appendicitis: a proposed algorithm for timely diagnosis.. J Pediatr Surg..

[3] ( 2008). Renu K, Mahajan JK, Rao KLN. Perforated appendix in hernial sac mimicking torsion of undescended testis in a neonate.. J Pediatr Surg..

